# High prevalence of thyroid carcinoma in patients with insulin resistance: a meta-analysis of case-control studies

**DOI:** 10.18632/aging.203529

**Published:** 2021-09-22

**Authors:** Junyu Zhao, Qianping Zhang, Yupeng Yang, Jinming Yao, Lin Liao, Jianjun Dong

**Affiliations:** 1 Department of Endocrinology and Metabology, The First Affiliated Hospital of Shandong First Medical University and Shandong Provincial Qianfoshan Hospital, Jinan 250014, China; 2 Department of Endocrinology and Metabology, Shandong Provincial Qianfoshan Hospital, Cheeloo College of Medicine, Shandong University, Jinan 250014, China; 3 Division of Endocrinology, Dezhou Municipal Hospital, Dezhou 253000, China; 4 Division of Breast and Thyroid Surgery, Jinan Zhangqiu District Hospital of Traditional Chinese Medicine, Jinan 250200, China; 5 Division of Endocrinology, Department of Internal Medicine, Qilu Hospital of Shandong University, Jinan 250012, China

**Keywords:** insulin resistance, HOMA-IR, thyroid carcinoma, meta-analysis, case-control study

## Abstract

The association between insulin resistance and thyroid carcinoma is controversial. We conducted this meta-analysis of association between insulin resistance and thyroid carcinoma. There were 14 studies included in this meta-analysis. Random-effect model was used to merge the weighted mean difference value of fasting serum insulin level and the pooled effect shows that the level of fasting serum insulin is higher in patients with thyroid carcinoma than those of controls (1.88, 95% CI 0.87 to 2.90, P=0.0003). Random-effect model was used to estimate the pooled weighted mean difference and it shows that thyroid carcinoma patients have a higher level of homeostasis model assessment of insulin resistance (HOMA-IR) than patients without thyroid carcinoma (0.54, 95% CI 0.29 to 0.78, P<0.0001). Fixed-effect model with the odds ratio of insulin resistance shows that insulin resistance could increase the risk of thyroid carcinoma 216% compared with participants without insulin resistance (3.16, 95% CI 2.09 to 4.77, P<0.0001). In conclusion, insulin resistance might be a risk factor for thyroid carcinoma.

## INTRODUCTION

Over the past decades, the incidence of thyroid cancer has increased markedly worldwide. In the United States, the yearly incidence tripled during the past 30 years [1]. Moreover, in China it has became the fifth most common cancer [2]. This increment can be partly ascribed to the rapid development and widely use of sonography techniques. However, it can not explain all, especially macrocarcinoma. Nowadays, the known risk factors for thyroid carcinoma include the head and neck radioactive exposure, female, advanced age, iodine deficiency or excessive and family history of thyroid carcinoma [3, 4]. Unfortunately, most of these are ineluctable. Recently, some scientists proposed that insulin resistance or hyperinsulinemia might be associated with thyroid carcinoma [5–11]. The homeostatic model assessment of insulin resistance (HOMA-IR) was used to calculate and determine if insulin resistance. The specific calculation equation is as follows: HOMA-IR = Fasting Serum Insulin (μIU/ml) • Fasting Plasma Glucose (mmol/l) / 22.5. It has been shown that insulin resistance is significantly associated with a larger thyroid volume and higher prevalence of thyroid nodules [12]. The association between insulin resistance or hyperinsulinemia and thyroid carcinoma has been reported by some studies [5–11], while others did not found the relationship [13–17]. Whether insulin resistance or hyperinsulinemia is related to thyroid carcinoma is still inconsistent. So, this study plans to investigate the association between insulin resistance and thyroid carcinoma, thus propose insulin resistance as a risk factor.

## RESULTS

### Search results and characteristics of included studies

**Figure 1:**
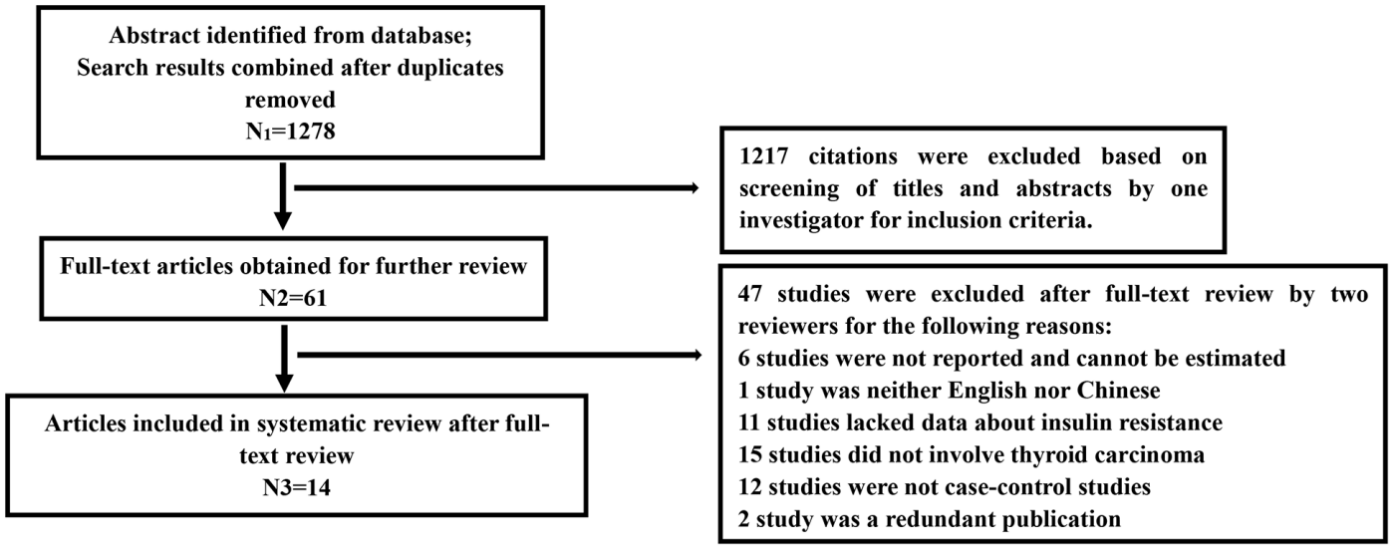
Flow chart of the systematic search process.

1278 relevant articles were collected after preliminary screening. Sixty-one articles were selected for full-text review after screening the abstract. Finally, 14 articles were included in this study. Figure 1 showed the details of systematic search process. Of these included 14 studies, three are published in Chinese [10, 11, 17] and the rest are all published in English [5–9, 13–16, 18, 19]. Four studies were conducted in Turkey and three in China. The rest countries including Italy, Argentina, Nepal, Korea and Iran, each has one study. Totally, there are 2024 patients with thyroid carcinoma in case group, and 1460 persons in control group which including healthy controls and patients with benign thyroid diseases. The sample size in case group ranges from 20 to 735 while 20 to 537 in control group. Patients in case group were diagnosed with histopathological. Among which, five studies reported the relationship between insulin resistance and PTC, and the rest seven reported the DTC. Table 1 summarized the detailed characteristics of these 14 studies.

**Table 1:** Characteristic of 14 included studies.

**First author, year**	**Country**	**Pathological type**	**Source of controls**	**Number of participants, n**		**Mean age, year**		**Female (%)**	
				**Cases**	**Control**	**Cases**	**Control**	**Cases**	**Control**
Massimo Giusti, 2008	Italy	DTC	Outpatients who had undergone thyroid surgery for benign thyroid diseases.	96	87	57.0±13.7	56.5±15.9	80	90
Jorge N. Rezzonico, 2009	Argentina	DTC	Normal thyroid function, normal thyroid gland palpation, negative titers of antithyroid antibodies and normal thyroid ultrasonography.	20	20	46.1±13.6	46.7±13.3	100	100
Ankush Mittal, 2012	Nepal	DTC	Normal healthy controls.	50	50	58.9±11.0	57.6±10.0	76	76
Mustafa Sahin, 2013	Turkey	DTC	Not mentioned.	344	116	45.5±11	44.9±8	84	85
Fevzi Balkan, 2014	Turkey	DTC	Euthyroid patients with nodular goiter who underwent surgery.	41	41	43.7±10.4	47.3±10.9	90	90
Musrafa Akker, 2014	Turkey	DTC	Subjects without a history of cancer, determined by thyroid ultrasonography not to have a thyroid nodule, or found to be cancer free following thyroid surgery.	93	111	50.2±12.2	36.9±9.8	85	57
Wang Dan, 2014	China	PTC	Benign thyroid nodule.	50	49	46.68±12.72	49.40±11.10	68	80
Min Jung Bae, 2016	Korea	PTC	No definite nodules or showed a typical nodule that was benign-looking in nature, and benign results after fine-needle aspiration cytology.	735	537	50.2±11.1	48.7±9.8	Not mentioned.	
Jiang Yanyan, 2016	China	PTC	Normal healthy controls.	358	290	44.0±11.8	43.9±14.0	78	66
Wo Xiaoyan, 2017	China	PTC	Benign thyroid nodule.	153	105	45.67±11.61	48.42±11.85	76	76
Bekir Ucan, 2017	Turkey	PTC	Age-, sex-, and body mass index-matched controls.	54	24	42.4±10	42.5±9	87	75
Zahra Heidari, 2017	Iran	DTC	Healthy euthyroid control participants were chosen with normal thyroid sonography.	30	30	34.4±12.7	34.1±12.8	80	80
Guo XY, 2019	China	PTC	Benign thyroid nodule.	153	105	45.7±11.6	48.4±11.9	76	76
Mele Chiara, 2019*	Italy	DTC	Benign thyroid nodule and healthy control.	30	27; 20	50.0 (41.0-58.8)	56.0 (53.5-65.0); 47.0 (37.0-62.5)	70	85; 75

DTC, Differentiated Thyroid Carcinoma; PTC, Papillary Thyroid Carcinoma; *Data are expressed as the median (interquartile range); The rest data are expressed as the mean ± standard deviation.

### Quality of included studies

Methodological quality of the case-control study was evaluated by NOS scores. Table 2 summarized the results of quality assessment of these 14 studies. Case control studies that achieved five scores or above were considered as a high quality study. As a result, all of these included studies were high quality.

**Table 2:** Quality assessment according to the Newcastle-Ottawa scale.

**First author, year**	**Section**	**Comparability**	**Exposure**	**Total**
Massimo Giusti, 2008	3	0	3	6
Jorge N. Rezzonico, 2009	3	1	3	7
Ankush Mittal, 2012	2	1	3	6
Mustafa Sahin, 2013	3	0	3	6
Fevzi Balkan, 2014	3	2	3	8
Musrafa Akker, 2014	3	1	3	7
Wang Dan, 2014	3	0	3	6
Min Jung Bae, 2016	3	0	3	6
Jiang Yanyan, 2016	3	0	3	6
Wo Xiaoyan, 2017	3	0	3	6
Bekir Ucan, 2017	3	1	3	7
Zahra Heidari, 2017	2	2	3	7
Guo XY, 2019	3	1	3	7
Mele Chiara, 2019	3	1	3	7

### Level of fasting serum insulin and thyroid carcinoma

**Figure 2:**
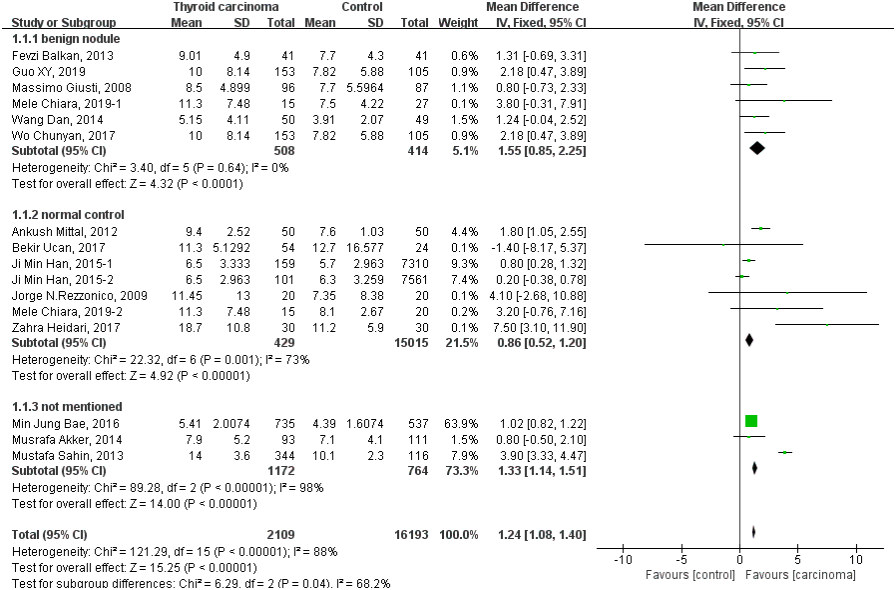
Forest plot of the fasting serum insulin level in patients with thyroid carcinoma.

Thirteen studies reported the level of fasting serum insulin [5–9, 11, 13–19]. Fixed-effect model was used to merge the WMD and pooled effect size is 1.24 (95% CI 1.08 to 1.40, *P*<0.00001), which showed that thyroid carcinoma patients have a higher level of fasting serum insulin than controls. Subgroup analysis with fixed-effect model was done by the different original of control participants that including benign nodule diseases, normal control and not mentioned. All the three subgroups show a significantly higher level of fasting serum insulin in the group of thyroid carcinoma (Figure 2). The pooled WMD in subgroup of benign nodule diseases is 1.55 (95% CI 0.85 to 2.25, *P*<0.0001) and no significant heterogeneity was detected (heterozygosity test, Chi^2^=3.4, *P*=0.64, *I*^2^= 0%). The pooled WMD values is 0.86 in subgroup of normal control (95%CI 0.52 to 1.20), whereas, a high heterogeneity was calculated (heterozygosity test, Chi^2^=22.32, *P*=0.001, *I*^2^= 73%). In conclusion, the level of fasting serum insulin in thyroid carcinoma patients is statistically significant increased compared to persons without thyroid carcinoma.

### HOMA-IR and thyroid carcinoma

**Figure 3:**
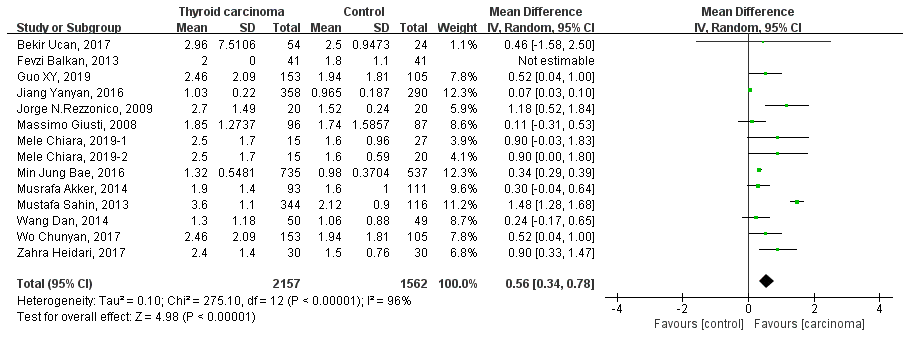
Forest plot of HOMA-IR in patients with thyroid carcinoma.

Thirteen studies analyze the relationship between HOMA-IR and thyroid carcinoma [5, 7–11, 13–19]. Random-effect model was used to estimate pooled WMD. The pooled WMD is 0.56 (95% CI 0.34 to 0.78, *P*<0.00001), whereas heterogeneity was detected between included studies (heterozygosity test, Chi^2^=275.10, *P*<0.00001, *I*^2^= 96%) and the results showed in Figure 3. That means a higher HOMA-IR is related to a high incidence of thyroid carcinoma.

### Insulin resistance and risk of thyroid carcinoma

**Figure 4:**
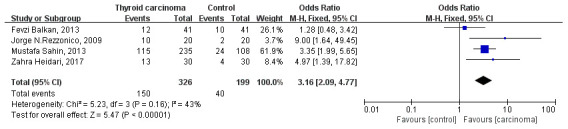
Forest plot of insulin resistance and risk of thyroid carcinoma.

Four studies reported the association of insulin resistance with the risk of thyroid carcinoma [5, 7, 9, 15]. Fixed-effect model was used to estimate pooled OR. The pooled OR is 3.16 (95% CI 2.09 to 4.77, *P*<0.0001) and showed in Figure 4. Whereas, a heterogeneity was detected between these four studies (heterozygosity test, Chi^2^=5.23, *P*=0.16, *I*^2^= 43%). In sum, insulin resistance increase the risk of thyroid carcinoma 216% compared with participants without insulin resistance.

### Publication bias

**Figure 5:**
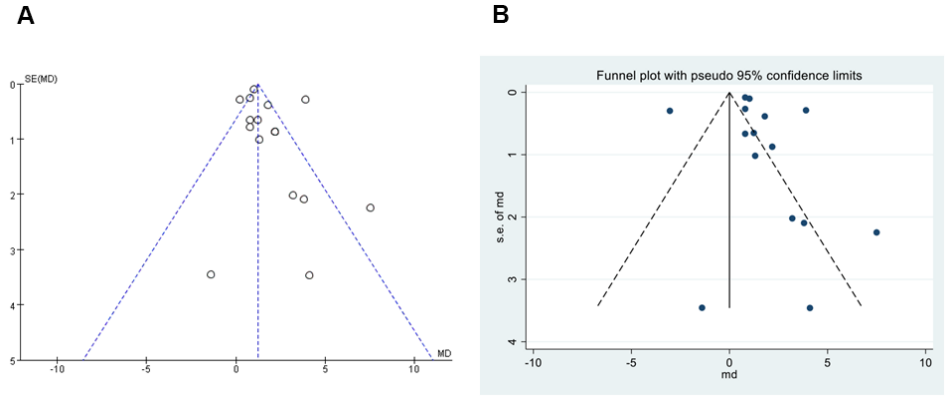
**Funnel plot of publication bias.** (**A**) Funnel plot, qualitative evaluation of publication bias, performed by Review Manager 5.3. (**B**) Egger`s test, quantitatively measurement of publication bias, performed by Stata16.0.

Funnel plot, a method for qualitative evaluation of publication bias, was done in this meta-analysis. All the studies are symmetry distributed at the top of the funnel plot made with review manage by visual observation (Figure 5A). We use stata16.0 software to detect publication bias, as shown in Figure 5B, the results show that there is no publication bias (Egger`s test *P*=0.565). According to the results showed above, there was no obvious publication bias in the included studies.

## DISCUSSION

The incidence of thyroid carcinoma has been markedly increased all over the word [2, 20, 21], therefore the risk factor of thyroid carcinoma attracting more and more attentions, especially the risk factors that can be prevented. Recently, insulin resistance [5, 7], obesity [22, 23], diabetic mellitus [24, 25] and other metabolic index have been found to be associated with higher incidence of thyroid carcinoma.

Whereas, the role of insulin resistance in carcinogenesis is still debatable [9, 15]. Previous studies have reported that the level of fasting serum insulin is higher in thyroid carcinoma patients than controls [5–11]. However, other studies did not show the same result [13–17]. We conducted this meta-analysis and finally concluded that insulin resistance and hyperinsulinemia might be a risk factor for thyroid carcinoma.

Insulin resistance has been reported to be a risk factor in many kinds of cancers, such as melanoma, endometrial, hepatocellular, colorectal, breast and even lung cancer [26–32]. In addition, it is also found to be associated with nodular thyroid disease [28]. Insulin resistance is generally paralleled by hyperinsulinemia. Insulin that beyond physiological dosage can promote thyroid cell growth, thus it can lead a carcinogenesis in patients with some benign thyroid diseases [33]. Furthermore, hyperinsulinemia can affect the metabolism of energy by increasing glucose uptake of cells, which can active some signal transduction pathway in cells, causing an excessive proliferation, and even promote the carcinogenesis and development of malignant tumors. The tumor markers in patients with hyperinsulinemia and normal control have already been detected. Moreover, it was found that proto-oncogene, such as human epidermal growth factor receptor-2 and B-cell lymphoma-2, were highly expressed, meanwhile tumor suppressor gene (p 53) was significantly decreased in patients with hyperinsulinemia [33]. In a word, it is reasonable speculate that insulin resistance or hyperinsulinemia may be the risk factor for thyroid carcinoma.

Nowadays, the mechanisms by which insulin exerts the carcinogenesis efficiency mainly focus on the study of insulin-like growth factor-1 (IGF-1). IGF-1, IGF-1 receptor and insulin receptors has been found in thyroid carcinoma, hyperinsulinemia and insulin resistance were also found to play a role of carcinogenesis [34]. Insulin shares structural homology with IGF-1 and thus it can bind to the IGF-1 receptor, thus participating in thyroid stimulating hormone -mediated proliferation of thyroid cells [35]. Liu et al. found that comparing with normal healthy population, the expression of IGF-1 and IGF-1 receptor in patients with follicular thyroid tumor, nodular goiter and papillary thyroid cancer were significantly higher [36]. It was supposed that the role of insulin in promoting the formation of thyroid nodule may be partially mediated by the proliferation effective of IGF-1. Moreover, hyperinsulinemia can induce mitotic and anti-apoptotic effects by acting on the IGF-1 receptor, protein kinase B, mammalian target of rapamycin and other signal transduction pathways [37]. From the previous research results, the mechanisms of hyperinsulin-induced carcinogenesis are not completely clear and needs further study.

It is reported that a reduced risk of thyroid nodules and decreased thyroid volume and nodule size were found when insulin resistance was decreased by metformin [38, 39]. And metformin can also inhibit the growth and migration of thyroid cancer cells, inhibit the self-renewal of tumor stem cells, and enhance the effect of chemotherapeutic drugs [40, 41]. Moreover, as an insulin-sensitizing agent, metformin was supposed to reduce the risk of thyroid cancer in Taiwanese type 2 diabetes mellitus patients [42]. Therefore, it is reasonable to suppose that insulin resistance or hyperinsulinemia was the risk factor for thyroid carcinoma.

There are some limitations in this meta-analysis: (1) results of insulin resistance were reported in only four studies, not all fourteen; (2) the cut-off level of insulin resistance were not consistent across these studies, which also caused some variation in results Meanwhile, the source of control groups in different studies were diversity, which may induce the heterogeneity and influence the results. Even so, we can still assume that insulin resistance is associated with an increased risk of thyroid carcinoma.

## CONCLUSIONS

The association between insulin resistance and thyroid carcinoma was summarized in this meta-analysis. Both high level of fasting serum insulin and insulin resistance are associated with increased risk of thyroid carcinoma. Thus, it can conclude that insulin resistance might be a risk factor for thyroid carcinoma. Due to the current limitations of this meta-analysis that described above, we believe that more prospective clinical studies with a large sample size may strengthen our conclusions. Meanwhile, more researches are needed to further elucidate the mechanism of insulin resistance causing increased risk of thyroid carcinoma.

## MATERIALS AND METHODS

### Searching progress

We searched for case-control studies that focus on thyroid carcinoma and insulin resistance simultaneously in the following databases: PubMed, Cochrane library, Sinomed, CNKI and Wanfang. The literature retrieval time limitation was: from the earliest data to 1 February, 2021. We used the following search terms for literature retrieval in the database: (“insulin resistance” or “IR” or “hyperinsulinemia” or “hyperinsulinaemia” or “hyperinsulinism” or “hyperinsulinism”) and (“thyroid cancer*” or “thyroid neoplasm*” or “thyroid tumor” or “thyroid carcinoma*” or “differentiated thyroid carcinoma” or “DTC” or “Papillary thyroid carcinoma” or “Thyroid carcinoma, papillary” or “PTC” or “Thyroid cancer, follicular” or “FTC” or “Thyroid Carcinoma, Anaplastic” or “ATC” or “Thyroid cancer, medullary” or “MTC”). Reference to all articles which considered for inclusion and related reviews, systematic review, etc. are also hand searched. We also searched the clinical trial register centers (http://www.clinicaltrials.gov) for clinical studies on this topic. The literature search was restricted to published (databases) or publicized (clinical trial register centers) results.

Inclusion criteria for this meta-analysis: (1) study that published in English or Chinese language; (2) study focused on the association between insulin resistance or hypersinulinemia and thyroid carcinoma; (3) study type was a case-control study; (4) at least one of the three outcomes was reported: the level of fasting serum insulin, HOMA-IR and the incidence of insulin resistance in patients with thyroid carcinoma. Articles that do not meet the inclusion criteria above will not be included in this meta-analysis.

### Study selection and data extraction

Two authors screened the literature and extracted the data independently. If there is any disagreement, the two authors will discuss it and decide. If discussions fail to resolve the doubt, a third, more experienced author (corresponding author) decides finally. The following information was extracted from the included studies: (1) characteristic of populations, including the pathological type of thyroid carcinoma, source of controls (benign thyroid diseases, healthy control, etc.), mean age and gender; (2) the results, including the fasting serum insulin level, HOMA-IR and the incidence of insulin resistance in patients with thyroid carcinoma.

### Methodological quality assessment

Newcastle-Ottawa Scale (NOS) was used to assess the methodological quality by two authors independently. If any disagreement, discuss and redecide. In the category of “Selection” and “Exposure”, each numered item can scored one star if the study meet the condition. While two stars can be got in the category of “Comparability”. Nine score is highest and shows a highest quality. This study has been conducted according to the PRISMA guideline and we have registered in INPLASY website, the registration number is INPLASY202180043 and the DOI number is 10.37766/inplasy2021.8.0043.

### Statistical analysis

The outcomes include the level of fasting serum insulin, HOMA-IR, the incidence of insulin resistance in patients with thyroid cancer. Fixed-model or random-model was performed by weighted mean difference (WMD), standardized mean difference (SMD) and 95% confidence intervals (CI) for continuous variables. Fixed-model performed by computing odds ratio (OR) and 95%CI for dichotomous variables. The heterogeneity of the included studies was evaluated by calculating I^2^. The analyses were performed by Review Manager 5.3 (Cochrane Collaboration, United Kingdom, http://www.cochrane.org) and STATA.
